# Two devices in one channel procedure for difficult cannulation due to periampullary diverticulum using a new duodenoscope

**DOI:** 10.1055/a-2612-3614

**Published:** 2025-07-02

**Authors:** Yusuke Takasaki, Yasuhisa Jimbo, Ippei Ikoma, Yusuke Yamaguchi, Daishi Kabemura, Sho Takahashi, Hiroyuki Isayama

**Affiliations:** 1 73362 Department of Gastroenterology, Graduate School of Medicine, University of Juntendo, Tokyo, Japan; 2 Division of Gastroenterology, Department of Medicine, Faculty of Medicine, Faculty of Medicine, Chulalongkorn University, Bangkok, Thailand

The two devices in one channel (2D-1C) procedure have been reported as useful in cases of difficult cannulation due to a periampullary diverticulum 1 2 . However, a limitation of this technique is that inserting two devices into the narrow working channel can reduce maneuverability. In this report, we describe the successful use of the 2D-1C procedure with a new duodenoscope (ED-840T, Fujifilm) featuring a larger 4.5-mm working channel than that of conventional duodenoscopes, resulting in improved maneuverability.

An 80-year-old woman undergoing chemotherapy for pancreatic cancer with liver metastasis was admitted to our hospital with obstructive jaundice and cholangitis. We attempted bile duct cannulation using the new duodenoscope and a catheter (MTW catheter; ABIS), but this was unsuccessful because of duodenal deformation caused by pancreatic head cancer and the presence of a periampullary diverticulum. As a result, we were only able to approach the papilla in the push position. We then attempted the 2D-1C procedure using small forceps (SpyBite Max; Boston Scientific Japan) ( Video 1 ). Despite working in the push position – which typically increases friction between devices within the endoscope – we were able to insert both devices without resistance. The forceps were used to grasp the anal side of the papilla and push it toward the scope ( Fig. 1 ). This allowed successful wire-guided cannulation of the bile duct using the catheter ( Fig. 2 ). After performing endoscopic sphincterotomy, we put in place a metal stent (Dumbbell ComVi, 10 mm × 6 cm; Century Medical) ( Fig. 3 ).

Two devices in one channel procedure for difficult cannulation due to periampullary diverticulum using a new duodenoscope.Video 1

**Fig. 1:**
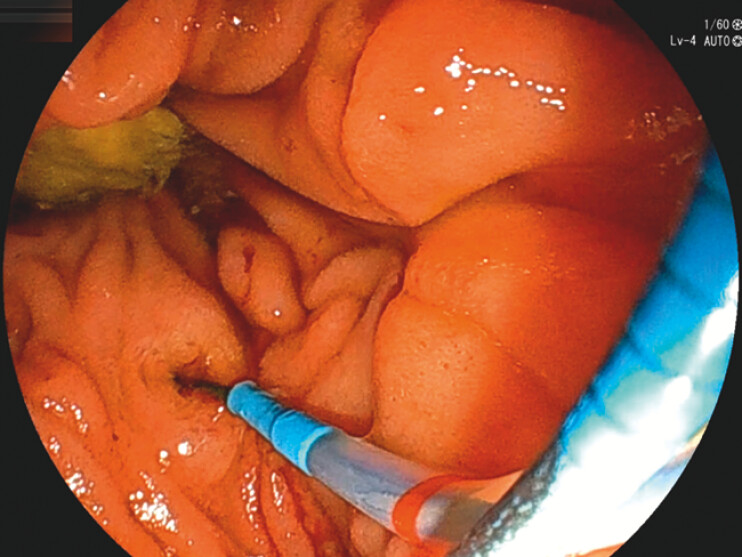
The papilla was grasped with small biopsy forceps and pulled toward the anal side. The cannula was then inserted into the bile duct using the wire-guided cannulation method.

**Fig. 2:**
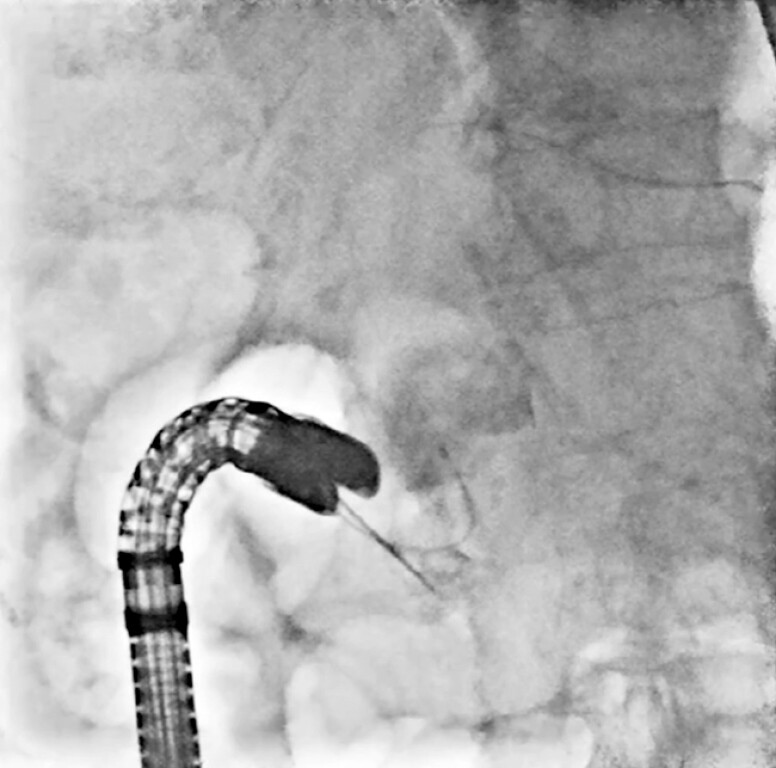
Fluoroscopic image showing bile duct cannulation performed using the two devices in one channel method in the push position.

**Fig. 3:**
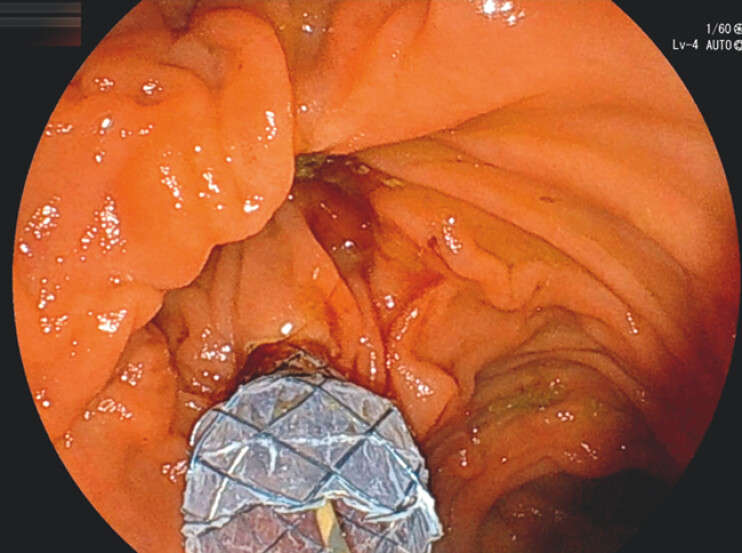
Placement of a metal stent (Dumbbell ComVi, 10 mm × 6 cm; Century Medical).

The new duodenoscope has a larger working channel and is well-suited for the 2D-1C technique.

Endoscopy_UCTN_Code_TTT_1AR_2AC

## Notes

Endoscopy E-Videos https://eref.thieme.de/e-videos
*E-Videos* is an open access online section of the journal *Endoscopy* , reporting on interesting cases and new techniques in gastroenterological endoscopy. All papers include a high-quality video and are published with a Creative Commons CC-BY license. Endoscopy E-Videos qualify for HINARI discounts and waivers and eligibility is automatically checked during the submission process. We grant 100% waivers to articles whose corresponding authors are based in Group A countries and 50% waivers to those who are based in Group B countries as classified by Research4Life (see: https://www.research4life.org/access/eligibility/ ). This section has its own submission website at https://mc.manuscriptcentral.com/e-videos .
